# On the Development of a Digital Twin for Underwater UXO Detection Using Magnetometer-Based Data in Application for the Training Set Generation for Machine Learning Models

**DOI:** 10.3390/s23156806

**Published:** 2023-07-30

**Authors:** Marcin Blachnik, Roman Przyłucki, Sławomir Golak, Piotr Ściegienka, Tadeusz Wieczorek

**Affiliations:** 1 Department of Industrial Informatics, Silesian University of Technology, 44-100 Gliwice, Poland; roman.przylucki@polsl.pl (R.P.); slawomir.golak@polsl.pl (S.G.); piotr.sciegienka@polsl.pl (P.Ś.); tadeusz.wieczorek@polsl.pl (T.W.); 2 SR Robotics Sp. z o.o., Lwowska 38, 40-389 Katowice, Poland

**Keywords:** digital twin, UXO, unexploded ordnance, training data set, magnetic field, FEM, magnetometer, finite element method, AUV

## Abstract

Scanning underwater areas using magnetometers in search of unexploded ordnance is a difficult challenge, where machine learning methods can find a significant application. However, this requires the creation of a dataset enabling the training of prediction models. Such a task is difficult and costly due to the limited availability of relevant data. To address this challenge in the article, we propose the use of numerical modeling to solve this task. The conducted experiments allow us to conclude that it is possible to obtain high compliance with the numerical model based on the finite element method with the results of physical tests. Additionally, the paper discusses the methodology of simplifying the computational model, allowing for an almost three times reduction in the calculation time without affecting model quality. The article also presents and discusses the methodology for generating a dataset for the discrimination of UXO/non-UXO objects. According to that methodology, a dataset is generated and described in detail including assumptions on objects considered as UXO and nonUXO.

## 1. Introduction

In the world, and especially in the geographical area of Europe, the problem of munitions, chemical weapons, and shipwrecks with fuel in tanks dumped in water reservoirs is becoming an increasingly urgent problem to solve. Particularly after World War II, munitions and chemical weapons remaining after the war, which various countries wanted to get rid of in the cheapest possible way, were sunk en masse. It is estimated that in the Baltic Sea, there may be at least 40 thousand tons of the war remnants mentioned above [1]. Although two main areas for dumping weapons were selected here, the Gotland Deep and the Bornholm Deep, a significant part of the weapons was dumped in unidentified places. Currently, due to progressing corrosion, the threat of the unsealing of weapons and release of poisonous substances into the environment, and the intensive installation works carried out in maritime areas, the development of effective and efficient methods to identify dangerous objects is required.

The task of searching for dangerous objects in the aquatic environment, in particular unexploded ordnance (UXO), requires scanning the surveyed area with various types of sensors (magnetometers, sonars, video cameras, although in this article we focus only on magnetometers). In the next step, specialists analyze the collected data and mark places containing potentially dangerous objects. These sites are subject to further inspection or extraction work carried out by sapper divers. Their work is very expensive because of the limited number of such experts, as well as the limited time possible to spend under the water.

**Figure 1:**
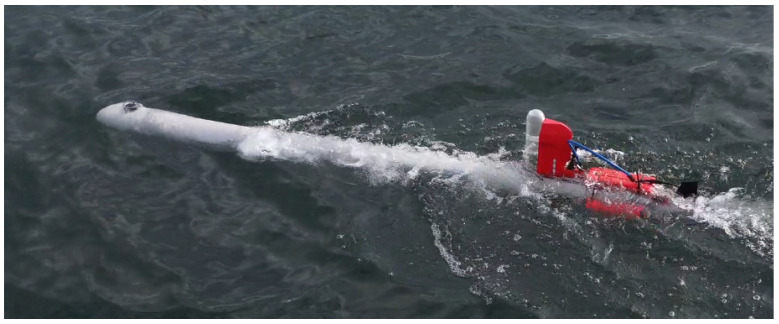
Autonomous underwater vehicle developed for UXO detection.

The scanning process is often conducted by a set of magnetometers (usually two or more) mounted on the wing pulled behind the ship or mounted on a formation of the AUVs scanning the interest area in parallel. An example of an AUV is a self-constructed vehicle shown in Figure 1.

In order to optimize the above process, one of the possibilities is to apply machine learning methods for the automatic identification of dangerous objects in particular UXO [2,3]. Considering that the scanned areas are usually calculated in a couple of square kilometers or more, automatization of data analysis should speed up the process. Even more, it is often possible to surpass the performance of human experts by more accurate classification of underwater objects by the use of machine learning models, as indicated in [4]. However, there is one potential weakness in the application of machine learning methods, which is the requirement for a large amount of good-quality training data. This issue is becoming even more important for the marine environment, where the collection of such data is associated with very high costs, as well as being labor-intensive and time-consuming. Additionally, the popular approaches for machine learning applications for UXO detection are based on electromagnetic induction (EMI), which is not applicable in underwater exploration, where the electromagnetic field is strongly attenuated. In that scenario, only magnetometers, which measure the change of earth’s magnetic field based on the magneto-static effect, are useful. Although, they are not as adept at correctly identifying UXO from non-UXO objects [5]. This is caused by several factors and additional requirements, e.g., if one wants to use historical data from real physical scans of various areas, then it is only possible for similar latitudes. A change in latitude causes a change in the magnetic inclination, and thus a different magnetic image is observed. In addition, a change in the orientation of the projectile, e.g., a change in spatial orientation angles, will result in a different magnetic image, or changing the angle of the path of the magnetometer over the projectile by a few degrees can cause a significantly different magnetic image. Thus, collecting the appropriate empirical data for training the AI models becomes physically very burdensome or even impossible.

In order to overcome the problem of the lack of magnetometer-based data, we propose building a full-featured digital twin of the UXO objects and their surroundings. A similar approach was previously attempted in [6], but the authors ignored remanent magnetization, which is particularly important within the water environment. Additionally, in [6], the authors compared a commonly used dipole model of UXO objects, which did not provide sufficiently accurate results. Thus, in our work, we propose a UXO model based on a digital twin that incorporates remanent magnetization using the finite element method. This approach more accurately simulates the shape of real ammunition and its real signals. The quality of the model is calibrated using real measurements. Obtaining a validated model allows us to conduct a series of simulations to generate full training datasets containing simulations of the passage of a group of probes equipped with magnetometers over potential UXO-based scans. However, this approach faces several issues:

1. Achieving the agreement between the synthetic model (digital twin) and real-live data—that is the match between the data recorded by the real magnetometers and the data recorded in the virtual environment.
2. Meshing problem considering the size of the 3D virtual environment that is a few meters large and the size of the UXO object that has walls a few millivoltmeters thick

In this article, we provide a detailed explanation of the process of developing such a digital twin and how we overcome the issues presented above. It must be noted that in the physical experiments conducted to validate the numerical model, we used a surrogate UXO object, which was a pipe with a length of 343 mm and 75 mm of the diameter corresponding to the typical ammunition. The term typical ammunition is defined as ammunition considered dangerous while conducting work at the bottom of water reservoirs. The pipe was used for safety reasons and allowed us to carry out the experiments without the supervision of sappers. Similarly, in numerical experiments, the UXO object was modeled with a pipe, allowing us to confirm and validate the model. When the numerical model is calibrated, we use it for the generation of a dataset for machine learning-based UXO/non-UXO classification. Therefore, in the virtual environment, when generating the training set, we model not only the pipe under consideration but also other pipes of different lengths and diameters that are too big or too small for UXO and other objects such as ferromagnetic sheets, rings, etc.

The novelty of this article is:

1. Introducing digital twin for UXO classification for magnetic data including remanent magnetization
2. Empirical verification of the digital twin using data from physical experiments obtained using magnetometer sensor
3. Introducing a means of computational complexity reduction without sacrificing the results obtained from the digital twin
4. Identifying important properties of the digital twin, which need to be implemented in order to achieve high comparability between physical data and the numerical model
5. Introducing a means of creating a dataset with multiple parallel virtual probes

The structure of this article is as follows: In Section 2, we provide the state-of-the-art in UXO identification and classification, including digital twin models. Then, in Section 3, we describe the mathematical model of the virtual environment, also introducing simplification of the model. In Section 4, experiments comparing the digital twin and the physical model are discussed. The results of the experiments are provided in Section 5. Section 6 describes the details of how we created the training set. Section 7 concludes the manuscript and summarizes the obtained results.

## 2. Related Work

There are several topics that are important in terms of UXO classification. The first is the development of better sensors, then the processing and modeling of recorded signals, and finally the development of the final classification model. Below, we discuss each of these topics.

### 2.1. Sensors Development

Sensors used for UXO detection are under rapid development, and new features and enhancements are added to the standard method. For example, in [7], the authors indicate that the source of the noise in the transient electromagnetic sensors is mainly caused by the receiving coil where the noise is dominated by the internal thermal noise of the damping resistor. Reducing the bandwidth of the system and increasing the size of the coil effectively reduces internal noise. Another group of sensors is constituted by magnetometers, where two are very popular: the fluxgate magnetometers [8], which have the advantage of delivering a three-dimensional vector of the magnetic field, and optically pumped magnetometers (OPM), which give more information at greater depth/height [9], although they return only the module of the signal. The OPMs are also under development and some recent developments include high-power off-resonant optical pumping; Mz configuration, where the pumping light and the magnetic field of interest are oriented parallel to each other; and the use of small alkali metal vapor cells of identical properties in integrated array structures. Another example of OPM optimization can be found in [10], where, after sensor optimization, the authors obtained a radio-optical cesium magnetometer sensitivity of 82.5 fT/$\sqrt{\mathrm{Hz}}$, a resolution within 1 pT to an external magnetic field change, and a noise fluctuation of 0.2 pT for an integration time of 1 s.

### 2.2. Signal Processing, UXO Modelling

In addition to the sensors, an important stage is the recorded signal processing. There are several approaches to UXO identification. A nice overview can be found in a report [11] in which the authors provide general approaches. Among them, they identify two main approaches. The first method is based on fitting measured magnetic data, neither TDEM nor FDEM, to a parametric model, and then using the recovered model parameters to classify the anomaly as UXO or non-UXO. Here, usually, a magnetic dipole model is used as a reference. The second approach is based on matching the measured data to the signature of known UXO and other objects. The authors also briefly discuss an approach based on SVM machine learning classification using the parameters obtained after matching the signals to the model. The properties of the dipole model were extensively evaluated by many researchers. For example, in [6] the authors study the properties of the physical dipole, which are then compared favorably with alternative models, including the limited cases of prolate spheroids and other shapes. Additionally, in the work, the authors critically review the explicit modeling of the demagnetization properties of magnetic materials. A more advanced study on UXO numerical models was conducted in [12] where the authors compared the prolate spheroid model with two more realistic UXO geometries using the finite element method. The results obtained showed that the calculated dipole moment response for complex models that resemble actual UXO is up to 50% higher than the dipole moments for the prolate spheroid model. They also found that altering the shape of a model from a prolate spheroid to a complex shape has a greater effect on dipole moment than maintaining the same shape and altering the volume. Finally, they found that complex models more closely match actual field data than prolate spheroid models. An application of the recorded signals to the fitted model can be found in many works. For example, in [13], the authors used a Normalized Surface Magnetic Source (NSMS) model and a variant of the simple dipole model to the data recorded using electromagnetic induction (EMI). For discrimination, the authors used two sets of parameters: intrinsic parameters associated with the size, shape, and material composition of the target; and extrinsic parameters related to the orientation and location of the anomaly. They found that the discrimination performance significantly depends on the mathematical models: single dipole, multidipole, and NSMS. In particular, when the noise is low and the UXO is isolated, the basic methods work well, but with noise and multiple targets placed close to each other, the complete method is more attractive. Similar work can also be found in [14,15]. In the last, the authors tested the spheroid model of UXO objects and analyzed how the object behaves in terms of the height of the recorded magnetic field and the caliber of ammunition. The authors have also studied how shock demagnetization behaves.

Another issue with UXO modelling is the problem of remanent magnetization, where it is often assumed that, as a result of the hitting shock, a demagnetization occurs. However, many authors suggest that remanent magnetization cannot be ignored and is very important. In particular, as indicated in [16], the remanent magnetization may remain. Moreover, the conducted experiments indicated that the opposite effect can occur, that the missile initially started with a very small remanence acquired a magnetic remanence in the direction of the inducing field at the time of impact, and the magnetic remanence is stable for time scales of up to one thousand years. Finally, it must be noted that in underwater research, many dangerous objects were sunk; therefore, shock demagnetization cannot be assumed and needs to be included in the model.

### 2.3. UXO Classification

UXO classification can be approached in various ways. One of the popular methods is solving the inverse problem. For example, in [17], the authors considered two inversion approaches: cooperative or constrained inversion; and (2) joint inversion. Cooperative inversion is the process of using inversion parameters from one dataset to constrain the inversion of other data. In a true joint inversion, the target model parameters common to the forward models for each type of data are identified, and the procedure seeks to recover the model parameters from all the survey data simultaneously. Besides the inversion approaches, a key element is the direct inverse algorithm. For example, this topic was covered in [18], where the eigenvector decomposition of the magnetic gradient tensor was used to locate dipole-like magnetic sources, allowing automatic detection of dipole-like magnetic sources without estimating the magnetic moment direction. A similar approach has also been discussed in [19], where the authors proposed a new algorithm with a magnetic gradient tensor and singular value decomposition (SVD) to estimate the target position and characterization quickly and accurately.

Another group of methods focuses on the use of electromagnetic data and the application of machine learning models. This idea is broad and influences not only military applications but also other areas of magnetic image processing [20]. However, in terms of direct UXO detection, the authors of [5] point out that “(…) magnetic data can only provide limited information about intrinsic target properties (i.e., size and shape) and are rarely used to classify detected targets as UXO and non-UXO.” Therefore, most machine learning applications focus on EMI data. One of the early approaches to utilize machine learning methods, in particular the Probabilistic Neural Networks can be found in [21]. In [4], the authors discuss the concept of using linear genetic programming for UXO/non-UXO classification. A broader work that discusses many machine learning approaches can be found in [22], where the use of SVM and Probabilistic Neural Networks is discussed. Additionally, the authors discuss feature extraction techniques; in particular, they used a combination of a size and time-decay vector. As a result of their work, they developed the UXOLab software. The next example of utilizing a machine learning model is presented in [23], where the authors combine supervised learning such as SVM and neural networks with unsupervised learning such as Gaussian Mixture Modelling. As a feature space, they use features extracted from the EMI decay curves of the physics-based intrinsic, effective dipole moment, called the total Normalized Surface Magnetic Source (NSMS). They found that such a combination provides a reduction in the amount of required training data and allows for a convenient probabilistic interpretation of the classification.

A similar approach to ours can be found in [24], where the authors use total field magnetic responses obtained using the finite element method to train various classifiers. In particular, they used Random Forest, Support Vector Machine, and Neural Networks, additionally using several types of labels, where the based performance was obtained when the classes were derived from a multiclass self-organizing feature map (SOFM). Although, in their work the authors assumed the lack of remanent magnetization, which may be of particular importance in terms of the underwater classification. Another example of using machine learning methods in UXO classification was discussed in [2], where the authors used data obtained from ground-penetrating radar. The results obtained allowed them to achieve an accuracy that ranged between 89% and 92%.

## 3. The Digital Twin Model

**Figure 2:**
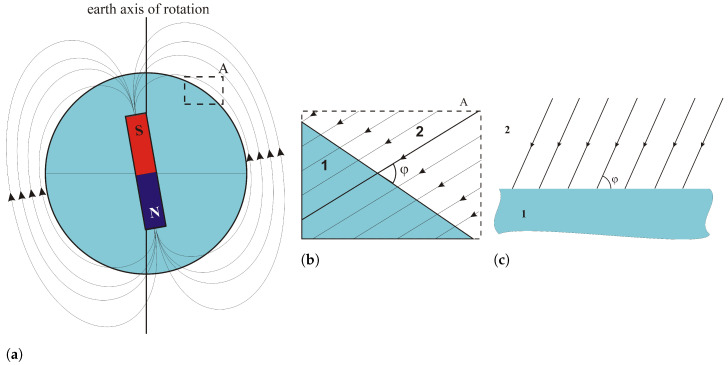
Earth magnetic field (**a**) global view, (**b**) local view of A detail (**c**) local view of A detail in classical perspective (earth surface horizontal); 1—earth; 2—air (or water); φ—magnetic field inclination.

As mentioned in the introduction, one of the techniques used to detect dangerous objects is the study of the distortion of the earth’s magnetic field. This is a passive technique and requires the use of magnetic field sensors. As known, the earth is the source of the magnetostatic field. The distribution of the field on a global scale changes intensively (Figure 2a), but on a local scale it can be assumed that the earth’s magnetic field is homogeneous, and the field lines are parallel to each other (Figure 2b,c).

**Figure 3:**
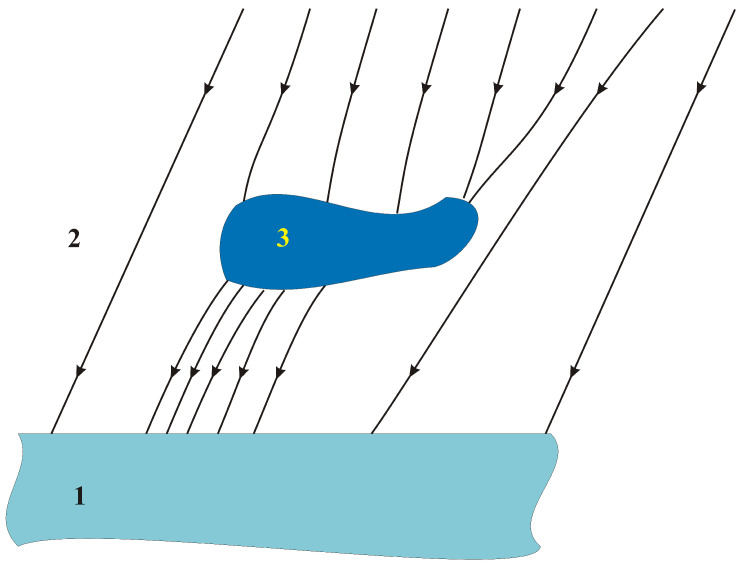
Distorted Earth magnetic field 1—earth; 2—the surroundings of the object (air, water); 3—magnetic object.

If the field force lines are not parallel, it means that there are objects (natural or anthropogenic) disturbing this distribution. To distort the distribution of the magnetic field (according to the Formula (5)), there must be a change in the magnetic permeability of the medium (change in magnetic permeability μ) or an additional magnetic field must occur (magnetization M). It follows that the technique of studying changes in the earth’s magnetic field allows the detection of ferromagnetic objects. Theoretically, diamagnetic or paramagnetic objects are also detectable. However, due to small differences in the value of relative magnetic permeability, the detection of paramagnetic and diamagnetic materials in the aspect analyzed in this article has no practical significance. An example of distortion of the magnetic field force lines by a ferromagnetic object is shown in Figure 3.

In order to create a digital twin of the UXO object, two elements are needed; these are a proper mathematical model of the system and an environment which will be used to apply this model.

### 3.1. Mathematical Formulations

In terms of the mathematical model, it was assumed that within the virtual environment, the Earth’s magnetic field is homogeneous with an inclination of 67°. Within this environment appears an isolated ferromagnetic object, in particular, a UXO object with known properties such as magnetic permeability. Initially, we assumed no additional magnetization of the twin object; however, during the experiments, the object was extended with the magnetization vector. More formally, the equations allowing the analysis of the static magnetic field in the absence of current are derived from Maxwell’s equations. From Gauss’s law for a magnetic field in the form (1) and Ampere’s law in the form (2) [25].

(1) ∇·B=0

(2) ∇×B=0

where:

B—magnetic flux density.

Supplemented by the equation describing the magnetism in materials in the form (3) and the constitutive Equation (4) [26],

(3) $$B=B_{0}+\mu_{0}M$$

(4) $$B=\mu_{0}\mu_{r}H$$

where:

$B_{0}$—outside magnetic flux density,

$\mu_{0}$—the magnetic permeability of vacuum,

M—magnetization vector,

$\mu_{r}$—the relative magnetic permeability.

Finally, the equation describing the magnetostatic field without the presence of current for linear and isotropic media takes the form (5) [26]:

(5) $$\nabla \times \left(\frac{1}{\mu }B-M\right)=0$$

where:

μ—magnetic permeability.

### 3.2. Modelling Environment

The research presented in this article was based on a computational model prepared in the Gmsh-Getdp environment [27]. The Gmsh-Getdp software is a universal, open, still-developing numerical modeling environment that allows the creation of three-dimensional models of many physical fields. In this case, this program was used to model the static magnetic field.

In terms of the scale of the virtual environment, we needed to take into account the size of the scanned object and the properties of the magnetic disturbance. As indicated in [28], the greatest problem of the Baltic Sea, in terms of numbers, is artillery ammunition. Over 40 times more artillery ammunition was sunk than other dangerous objects, for example, 400,000 pieces in the Bornholm Deep. Typical World War II artillery shells ranged in caliber from 75 mm to 150 mm. Considering that both UXO and non-UXO objects were to be simulated, the permissible diameter/caliber range was 20 mm—a non-UXO object (not dangerous) up to 300 mm, where the acceptable, largest UXO objects had a diameter of 150 mm, and objects over 150 mm were treated as non-UXO. The range of 5 mm to 15 mm was adopted as the wall thickness of the projectile according to [29,30].

Secondly, the volume of the simulated environment needed to be determined. In general, it is assumed that the shorter the distance between scanned object and the magnetometer, the greater the visibility of object details [15], and, as indicated in [31], when the distance is above 2.5× the length of the scanned object, it begins to resemble the image of a magnetic dipole. When determining the selection of the scanning height, the information of magnetometer manufacturers is also important, which indicates that the maximum detection distance of an object weighing 2 kg is 4 m [32]. On the other hand, due to the safety of the AUV, it is advisable to operate as high as possible above the bottom, which is why the developed virtual model assumes a range of 1 m to 4 m above the tested object.

In the case of a model reproducing the environment in which the probes equipped with magnetometers move over the object at a distance preventing their damage and covering the area of the probes’ passage to measure the entire magnetic field disturbance, we encounter the problem of the difference in scales between the size of the entire modeled environment and the size of the recognized object. This means, above all, a problem with the generation of the mesh used by the finite element method. Such a mesh must reflect the geometry of the object to the extent that it ensures the correct determination of the magnetic field in the vicinity of the object through which the probes pass. At the same time, however, as you move away from the object, taking into account the physics of the magnetic field, the mesh density should be reduced so that the calculations of the EM field distribution can be carried out in an acceptable time based on the available power of the computing cluster. This is possible due to the decrease in the disturbance of the magnetic field as a function of the square of the distance from the object (approximately).

**Figure 4:**
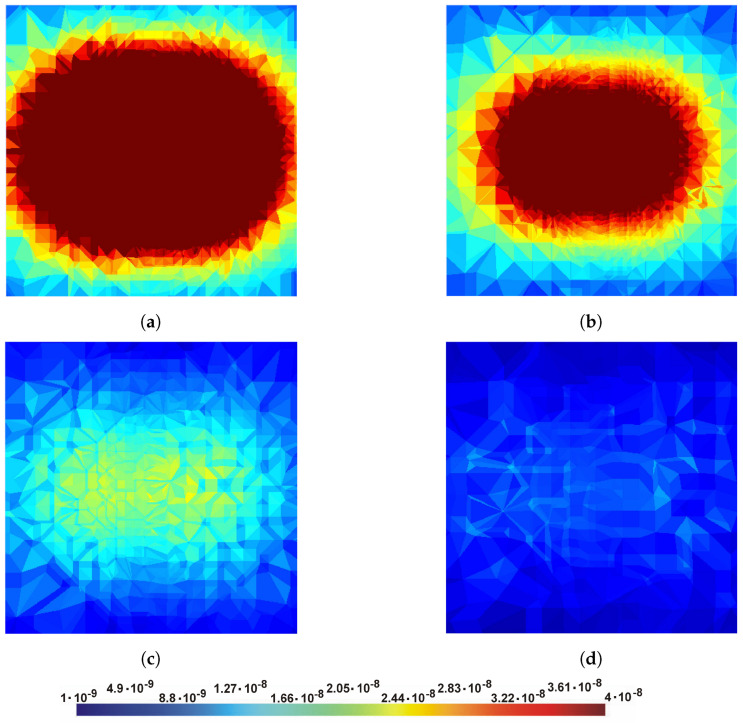
Exemplary relative (ΔB) change of the magnetic field at the following heights: (**a**) 1 m; (**b**) 2 m; (**c**) 3 m; (**d**) 4 m over the ferromagnetic object—plot from simulation, external dimension of the plot area is 6 m × 6 m.

Due to the typical, cone-shaped spread of the magnetic field disturbance, at a height of 1 m, a change in the magnetic field of 1 nT (the sensitivity of scalar cesium magnetometers is even pT) is detectable at a distance of approx. 8 m from the center of the projectile Figure 4a; at a height of 4 m, this distance is 6 m. For such assumptions, the computational model must cover an area of 8 m × 8 m.

**Figure 5:**
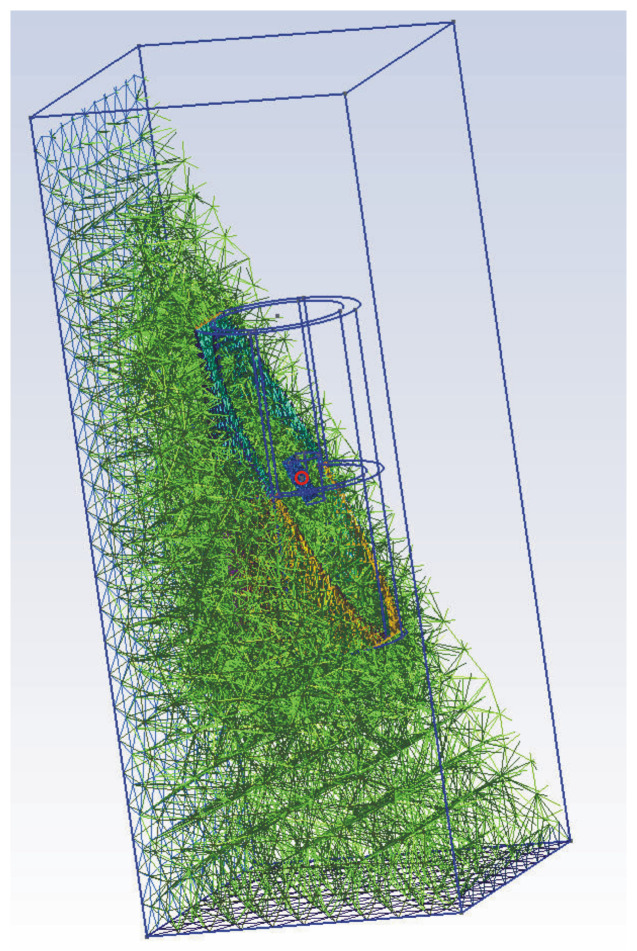
General view of the discretization mesh, the object in the center of the calculation area in red circle (almost invisible).

Such a large computing area in relation to the small wall dimensions of approx. 5 mm raises serious problems with correct model discretization. This manifested itself in two ways, either a very fine mesh, which guaranteed high accuracy of calculations at the expense of long calculation times, or in the other extreme case, a relatively coarse mesh (expanded in a classical way towards the outer borders of the calculation area), but then the calculation results at a great distance from the tested object were determined incorrectly. The compromise solution turned out to be an additional fineness of the mesh in the computational area of our interest Figure 5.

During the creation of meshes, we identified that proper results are obtained for mesh quality measures such as SICN (SICN—signed inverse condition number), Gamma (Gamma—inscribed radius/circumscribed radius), and SIGE (SIGE—signed inverse error on the gradient of FE solution) reaching values presented in Table 1. These values were used as a reference for all of the simulations.

### 3.3. Model Simplification

As previously indicated, the model encountered a problem with proper meshing that resulted in a very high density of mesh nodes within the walls of the pipe. This resulted in a long computation time.

To solve this problem, it was decided to perform preliminary calculations for simplified models, replacing the thin-walled object (pipe) with a cylinder. Two simplified models were considered: one is a cylinder with relative magnetic permeability identical to the relative magnetic permeability of the exact model, and the other simplified model, which is a cylinder of magnetic permeability equivalent to the magnetic permeability of the exact model. The equivalent permeability was determined based on the following Equations (6) and (7). The $\mu_{re}$—equivalent permittivity expresses volumetric equivalence.

(6) $$\mu_{rb}V_{b}=\mu_{re}V_{re}$$

(7) $$\mu_{re}=\frac{\mu_{rb}V_{rb}}{V_{re}}$$

where:

$\mu_{rb}$—base relative permeability (permeability of original object),

$\mu_{re}$—equivalent relative permeability,

$V_{rb}$—volume of original object,

$V_{re}$—volume of the substitute object.

**Figure 6:**
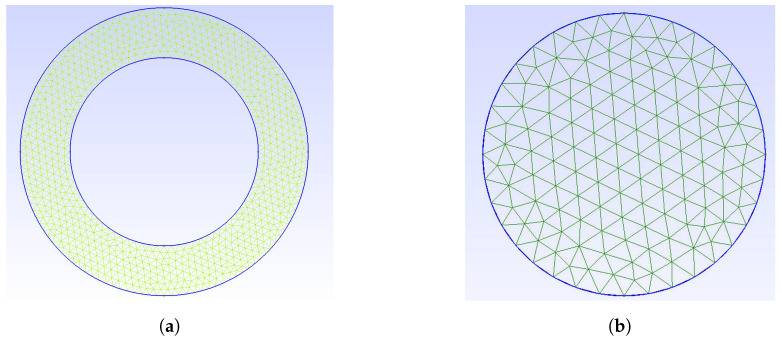
Discretization meshes (**a**) for a pipe (original object); (**b**) for a cylinder (substitute object).

The use of a cylinder as a model made it possible to maintain the correctness of the discretization mesh in the object area and reduce the number of mesh nodes. Exemplary meshes for a pipe and a cylinder are shown in Figure 6a,b.

During creation of the meshes, efforts were made to maintain similar measures of mesh quality for both models, mesh quality factors SICN, Gamma, SIGE only for objects are listed in Table 2, while mesh quality measures for the entire model along with the number of nodes are listed in Table 3. Due to the model simplification, we were able to reduce the computational complexity of the system. With a smaller number of nodes, there are fewer calculations required. The benefits of switching between a pipe and an equivalent cylinder are also shown in Table 3 in the last column.

### 3.4. Study of the Impact of Mesh Density on the Accuracy of Calculations

In addition to simplifications of the model, another classic problem for numerical modeling is the correctness of discretization of the computational model. A typical experiment was performed to check the influence of discretization on the accuracy of calculations. Calculations were made for both geometries (tube and cylinder), for three different mesh densities: low (reduced) mesh density (LD), reference density—for which simulation calculations were performed in this article (RD) and high mesh density (HD). Changes in mesh density were made only in the object area, efforts were made to maintain similar mesh quality measures. The data describing the experiment are presented in Table 4.

For the mesh with reduced density (LD) for the tube model, approx. four el per wall cross-section were assumed, for the reference mesh (RD), the number of elements on the wall cross-section was eight, and for the HD mesh, the number of elements on the wall cross-section was fifteen. For the cylinder model, for a mesh with a reduced number of elements, approx. nine elements on the diameter of the cylinder were assumed. For a mesh with a reference density for the cylinder model, approx. thirteen els on the diameter of the cylinder were assumed, and for the HD mesh, the number of elements on the diameter of the cylinder was twenty-two. To maintain comparable measures of mesh quality, the number of elements along the length of the object (both, tubes and cylinders) was also modified accordingly.

**Figure 7:**
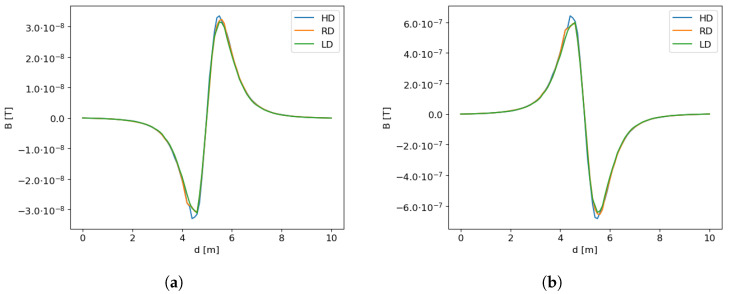
ΔB distribution for the N-S and E-W directions and different mesh density for the tube and cylinder model. (**a**) N-S direction and the tube model. (**b**) E-W direction and the cylinder model.

For three models differing in mesh density for each variant (tube, cylinder) (described above), a comparison of the obtained results was performed. The linear distribution of changes in magnetic induction (ΔB) in the directions N-S and E-W was adopted as a measure. Here, we simulated a path of a magnetic sensor passing 1 m above the object. The graphical comparison of the recorded signal, representing the influence of the grid quality is presented in the Figure 7a (N-S) and Figure 7b (E-W).

Additionally, we also measured the quality of the recorded signals using $R^{2}$ measure, which is defined as:

(8) $$R^{2}\left(y,\hat{y}\right)=1-\frac{SS_{res}}{SS_{tot}}$$

where *y* is the reference signal and $\hat{y}$ is the signal to be compared with the reference signal, and $SS_{res}=\sum_{i}{\left(y_{i}-{\hat{y}}_{i}\right)}^{2}$ is the sum of squares of residuals, and $SS_{tot}=\sum_{i}{\left(y_{i}-\bar{y}\right)}^{2}$ is the total sum of squares that is proportional to the variance of the data. As a reference value, we used the HD signal recorded when passing over the object and compared it with other signals recorded for RD and LD mesh. A comparison is presented in Table 5.

As the comparison shows, the deviations obtained between the variants differing in mesh density do not exceed 0.946 and, in the case of RD, 0.96. This proves that the grid selected for calculations (with reference density RD) is correct.

## 4. Digital Twin Validation Setup

The experiments consisted of three stages: the construction of a mathemat-ical-physical model, which has already been described in Section 3. Next, physical experiments were carried out involving the study of the UXO substitute with the use of magnetometers. The obtained results of the physical experiments were used to verify the quality of the mathematical model. In the next step, the mathematical model was improved in order to reduce the computational complexity, without losing its performance, as discussed in the previous section. Below, we present a description of each of these stages.

### 4.1. Empirical Experiments

**Figure 8:**
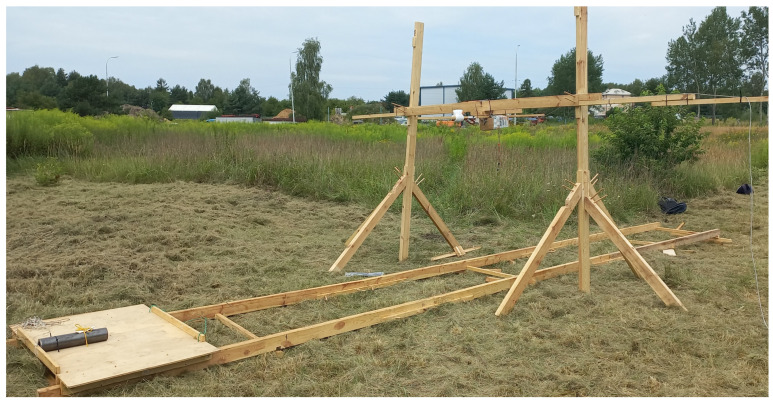
The magnetometer measuring station.

As already indicated, the empirical experiments were conducted using a pipe 343 mm long and 75 mm in diameter. Because the water environment does not affect the magnetic field, we conducted the experiments out of the water, in an environment free of magnetic sources other than the natural Earth magnetic field. To ensure a space free of ferromagnetic objects other than the UXO substitute, the construction of the measuring station was made of wood without any nails or screws, every part was assembled with wooden nails. At the test stand, the magnetometer or magnetometers were mounted on a wooden frame above the test object—similar to the mission with the use of AUV vehicles. The only difference was that, in the tested environment, it was the UXO object that moved. It was moved on the rails under the magnetometer, simulating a passage over the UXO object by the AUV containing the magnetometer. This allowed us to keep the computer station, which collects the measurements, separate from the measuring station. The magnetometers were hanged on 1.4 m above the rails, and the length of the rails was 7 m. The magnetometer was located in the middle of the rails. A view of the measuring station is depicted in Figure 8.

**Figure 9:**
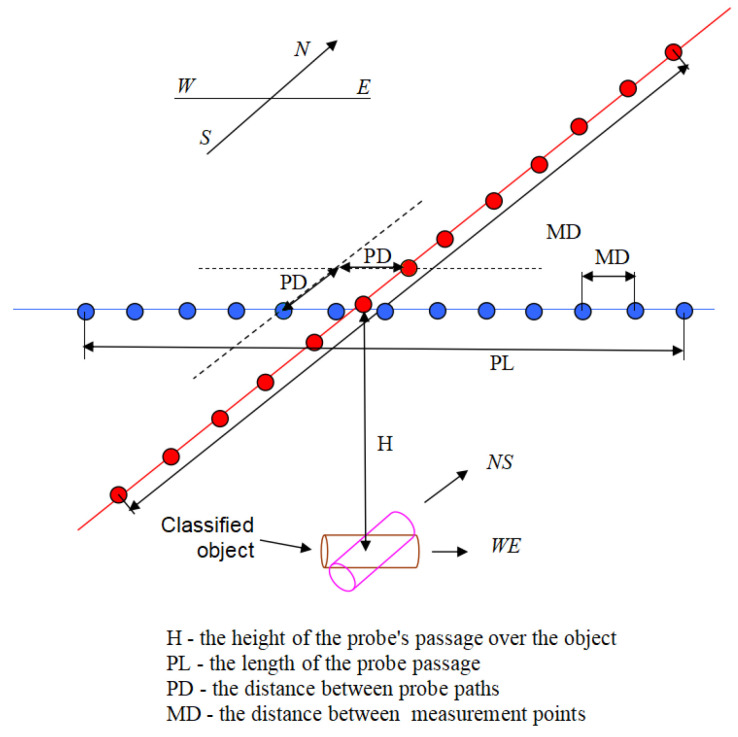
Measurement scheme.

In the experiments, we used the Geometrics MFAM LCS1005S magnetometer. The configuration of the measuring environment was as follows: orientation sensors for heading error cancellation, run mode: Low Noise and Two Independent Sensors, sampling frequency 1 kHz, notch filter 50 Hz. The measurement scheme is shown in Figure 9.

For data collection, we used the LabView software package, and the obtained values were stored in CSV files. Next, the CSV files were loaded into a self-developed environment, which was developed using Python and popular libraries such as Pandas, Numpy, SciPy.

### 4.2. Digital Twin Experiments

The digital twin of the virtual environment was built on the basis of the Gmsh [27] and GetDP [33] packages. The first tool was used to generate a mesh of the objects and a mesh of the environment. The results are then stored as a file which is delivered as input to the GetDP, which is used for simulations. The simulation results, which are returned as a cloud of points coincided with the nodes of the mesh. Each node is described by a 3D vector with x, y, z components of the magnetic induction within the virtual environment.

In real applications, the magnetometers are often mounted on a wing pulled behind a ship, or a magnetometer is mounted on an AUV where an AUV formation (usually three or more) is conducting the measurements. In order to simulate such a magnetometer configuration, we used the point cloud and an interpolator to achieve measurements with a fixed sampling rate. In the experiments, we used Matlab’s *scatteredInterpolant* function with *natural, linear* parameters, independently for each magnetic field component. Next, a path of probing was set up, including the angle of sampling in relation to the N-S direction, the height of probing over the center of the ferromagnetic object, and the distance between particular magnetometers, assuming all sampling was conducted at the same height. Additionally, the model included the possibility of adding randomness to the probing. The randomness influences the distance between magnetometers, and height, which results from imprecise positioning within the water environment. In the conducted experiments the randomness was set to 0 because we wanted to obtain maximum agreement between the physical and mathematical model. The obtained probing route was delivered to the input of the interpolator, which returned sampled values of each magnetic field component.

It is important to note that the proposed approach is very efficient and, for given object orientation, allows for conducting multiple experiments with many probing paths.

### 4.3. Data Processing and Signal Comparison Process

**Figure 10:**
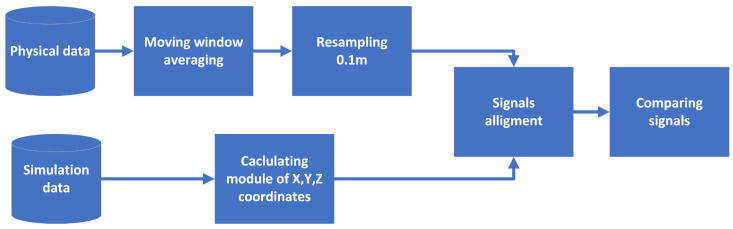
Data processing scheme.

The data recorded from numerical and empirical experiments were further processed using Python in order to compare the obtained results. The general scheme of data processing is presented in Figure 10.

First, the data recorded during the physical experiments were filtered, because they were noisy. In order to reduce the effect of the noise, the signal was sampled with a relatively high frequency (1 kHz). This allowed for the use of a moving average filter with a window size of five samples. That window size was enough to remove noise. Next, the signal was resampled with a frequency equivalent to the frequency used in the numerical experiments.

Next, the data obtained with the numerical simulations and physical experiments needed to be aligned to allow for comparison. The problem of alignment resulted from differences in the length of the probing route (11 m for the digital twin, and 7 m for the physical experiments) and the location of the magnetometer within the physical experiment, where it was not exactly in the middle of the route. The standard approach of signal alignment can be obtained using cross-correlation [34]; however, in the real scenario, often multiple signals needed to be aligned simultaneously—one per magnetometer. In our experiments, we used another measure, that is, the mean square error between signals y and $\hat{y}$, where the value of $n^{\prime }$ was under the subject of optimization.

(9) $$n^{\prime }=\underset{j=1..(N-M)}{argmin}\sum_{i=1}^{M}{\left(y_{j+i}-{\hat{y}}_{i}\right)}^{2}$$

where:

- *N*—the length of the signal recorded with a numerical model
- *M*—the length of the signal recorded with the physical model
- $y=[y_{1},y_{2},\ldots y_{M}]$—the values of the signal recorded using physical model
- $\hat{y}=\left[{\hat{y}}_{1},{\hat{y}}_{2},\ldots {\hat{y}}_{M}\right]$—the values of the signal recorded using digital twin
- $n^{\prime }$—the number of samples the signal y need to be moved forward or backward to align signal $\hat{y}$

After finding $n^{\prime }$, the signals were trimmed according to the shorter one. After the alignment of the signals, we calculated several performance measures, which allowed us to measure how well the numerical twin fits the real data. In the experiments, we used $R^{2}$ measure, which was already described, as well as the following:

- Root Mean Square Error (RMSE)—calculated as: $RMSE\left(y,\hat{y}\right)=\sqrt{\frac{1}{N}\sum_{i=1}^{N}{\left(y_{i}-{\hat{y}}_{i}\right)}^{2}}$
- Mean Absolute Error (MAE)—calculated as: $MAE\left(y,\hat{y}\right)=\frac{1}{N}\sum_{i=1}^{N}\left|y_{i}-{\hat{y}}_{i}\right|$
- Mean Error (ME)—calculated as: $ME\left(y,\hat{y}\right)=\frac{1}{N}\sum_{i=1}^{N}\left(y_{i}-{\hat{y}}_{i}\right)$
- Peak signal-to-noise ratio (PSNR) - calculated as: $PSNR\left(y,\hat{y}\right)=20\cdot \log_{10}\left(\frac{\max(y)-\min(y)}{RMSE(y,\hat{y})}\right)$

Where in the case of the PSNR we adapted this measure from image processing, it now identifies the ratio between the amplitude of the signal (max(y)−min(y)) to the level of noise that is the RMSE between the real and numerical signal expressed in logarithmic quantity using dB scale. Commonly, in image processing for compressed images the value of PSNR should be above 20 dB [35]. All these error measures were calculated assuming that the signal of the physical experiments is denoted as y and the one from numerical experiments is denoted as $\hat{y}$.

## 5. Evaluation of the Twin Validation Process

The first group of experiments was designed to achieve a full match between the digital twin and the physical experiments. First, we collected the data from the physical objects. For that purpose, the UXO object was scanned in different orientations and for different directions of scanning the object using AUV. In particular, we scanned in the following conditions:

1. UXO orientation—north-south, scanning route orientation north-south
2. UXO orientation—north-south, scanning route orientation east-west
3. UXO orientation—east-west, scanning route orientation east-west
4. UXO orientation—east-west, scanning route orientation north-south
5. UXO orientation—northeast-southwest, scanning route orientation east-west
6. UXO orientation—northeast-southwest, scanning route orientation north-south
7. UXO orientation—perpendicularly, scanning route orientation north-south
8. UXO orientation—perpendicularly, scanning route orientation east-west

For the main performance measures of the digital twin we used the $R^{2}$ and PSNR because both these measures refer to the signal and the level of noise, but the other measures discussed in the previous section are also provided in Table 6.

**Figure 11:**
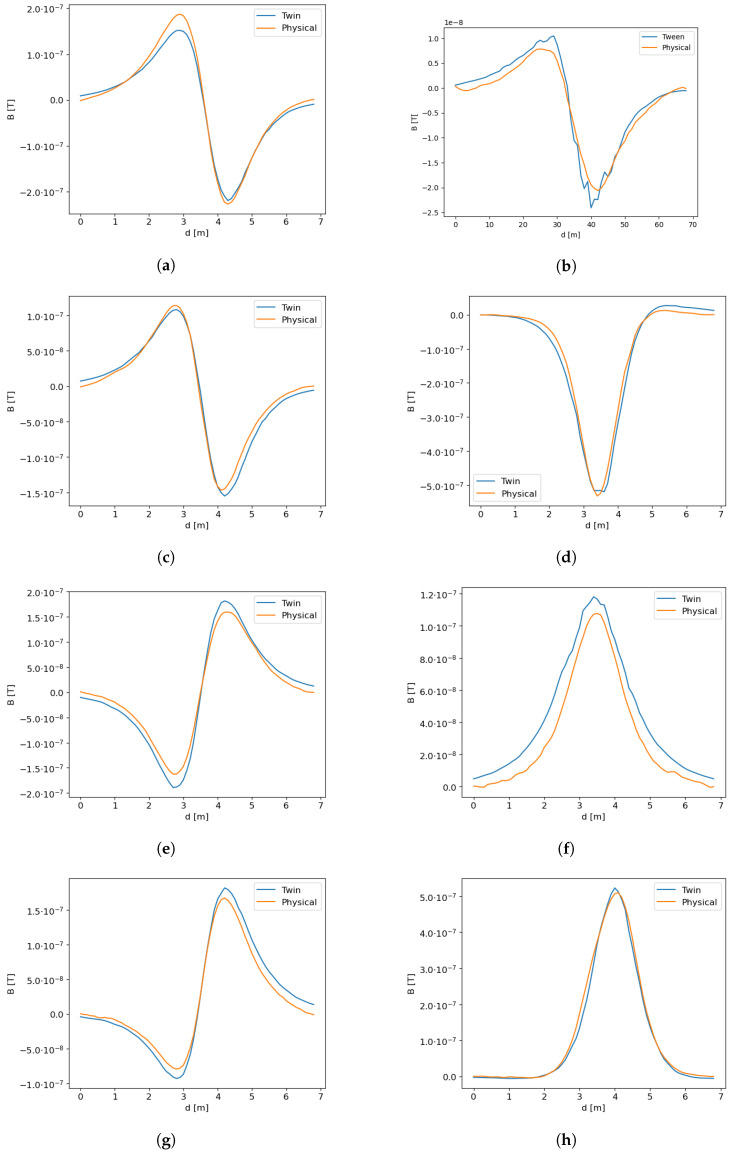
Visual representation of the match between the digital twin and the real magnetometer data. (**a**) N-S, N-S; (**b**) E-W, N-S; (**c**) NE-SW, N-S; (**d**) Perpendicularly N-S; (**e**) E-W E-W; (**f**) N-S E-W; (**g**) NE-SW, E-W; (**h**) Perpendicularly, E-W.

In the table, in almost all cases, the obtained performances of $R^{2}$ are on the level of 0.98 or 0.95, which indicates an almost perfect match. Similarly, the PSNR is in almost all cases above 20, and only in one case of N-S, E-W, both measures drop to 0.87 and 19 dB, respectively. This drop in quality results from the fact that when scanning an object perpendicularly to its longer, main axis, small changes in the angle result in significant changes in the recorded signal. For easier analysis of the results, we added a column denoted max−min, which indicates the amplitude of the signal, that is the difference between the maximum of the recorded physical signal and the minimum value of that signal. These results also indicate that the error rates of RMSE, ME and MAE are of one or two orders of magnitude smaller than the amplitude of the signal. All of the results indicate an almost perfect match. The quality of the match between the numerical model and the physical one is visualized in Figure 11, where both signals are plotted.

**Figure 12:**
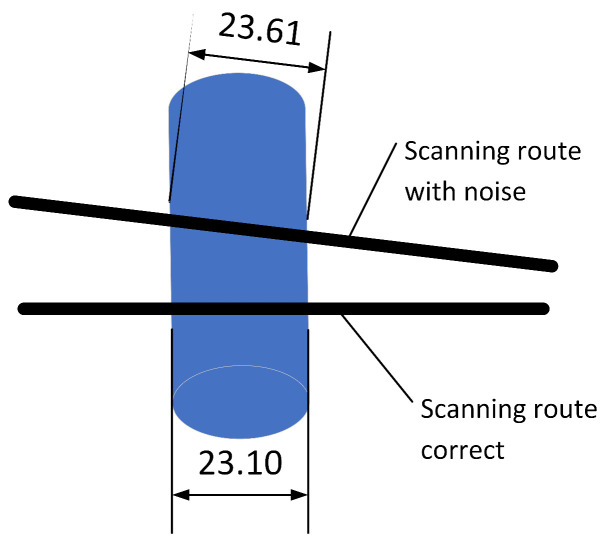
The source of the disagreement between the measurements and the model.

The only exception, that is $R^{2}=0.8724$, was obtained when UXO orientation was N-S and the scanning route was in E-W direction. This is also plotted in Figure 11f, where it can be observed that the shape is almost right, except for the magnitude of the signal. This can be explained by a small change in the orientation of the UXO object or E-W orientation that changes the active length of the object as shown in Figure 12.

Therefore, it can be concluded, that the digital twin matches the real physical signal very well.

The same calculations were also repeated for the cylinder substitute, which allows for a significant reduction of the calculation time. In that case, we obtained similar results as presented in Table 7. Here, a small, insignificant increase in all error rates can be observed $RMSE_{pipe}-RMSE_{cylinder}=3.0\times 10^{-10}$; therefore, we can conclude that there are no differences between both variants. This was achieved by treating the pipe and the cylinder as elements of a magnetic circuit whose basic feature used in detection is anisotropic (depending on orientation) reluctance. This gives the possibility of significantly simplifying the geometry of the object (replacing a hollow pipe with a cylinder), without a significant impact on the measurement obtained during the passage of the probes. Here, although the volume of the object changes, by keeping the reluctance fixed, we obtained identical results, and the reluctance is adapted by appropriately modifying $\mu_{r}$. Given that only the object’s anisotropic reluctance matters, the mesh parameters also have no significant effect on the simulated reading obtained, although the small difference in both RMSE may be caused by small differences in the obtained mesh.

Therefore, for the final training set creation, the cylinder substitute was used.

## 6. The Training Set Creation Procedure

The results presented above allowed us to conclude that the digital twin can be used for creating a training set for the machine learning purpose. Here, some additional benefits of the tandem of GMesh and GetDP appeared. These two tools are controlled by a script; therefore, it was very easy to automatize the process of generalization of the digital twin to produce many objects of different orientations. In particular, the great advantage of this solution is full control based on scripts over all stages of building the digital twin. In a single step of data generation, the following parameters were introduced to the model in the Gmsh/GetDP environment:

- strength and orientation (inclination) of the Earth’s magnetic field for the area for which experimental work was carried out (considered in the boundary conditions of the model),
- dimensions and orientation of the UXO and potentially other objects,
- material properties of this object (magnetic permeability, magnetization),
- mesh density.

**Figure 13:**
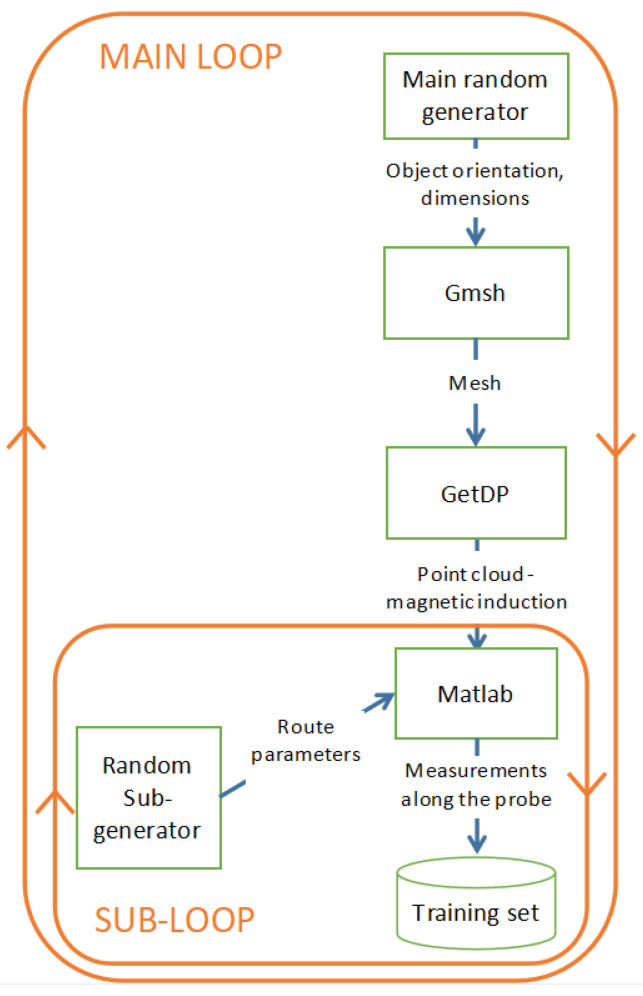
Scheme of the training set generation process.

Figure 13 shows the scheme of generation of the training set. The process consists of two loops. In the main loop, a random generator provides orientations and sizes of the modeled objects (in the range determined by the input parameters of the process). On this basis and the material parameters given at the input of the process, the Gmsh/GetDP model calculates the distribution of magnetic induction represented by a cloud of points. At this point, the inner subloop begins. The subgenerator randomizes the parameters of the probe route and, on this basis, the interpolator implemented in MATLAB simulates the magnetic field measurements during the passage of a group of probes. The scope of route randomization is also described by a set of input parameters of the generation process. After each flow simulation, another case is added to the training set.

For the final training set creation, most of the machine learning models need positive and negative cases (the so-called supervised learning). The final training set was obtained using a set of objects which are considered positive cases—UXO objects, and negative cases—non-UXO objects. The positive cases included substitute cylinders of predefined properties—the relation between the diameter and length of the cylinder, in particular, the length = four up to six diameters (that is the typical relationship between the length of the munition and its caliber). When the pipe has other properties it is considered a non-UXO object. In particular, as non-UXO objects, we considered munition that has a caliber that is too small, less them 70mm, because it does not cause significant danger for the works conducted on the bottom of the water reservoirs, and a caliber that is too large, >200 mm, and objects that are too long, which were considered as simple long pipes—not a munition.

Additionally, sheets of metal were also considered as non-UXO objects. They had a thickness of 10 mm to 30 mm and different lengths and widths. The length and width were selected to have a similar mass to the UXO objects. The constructed model also allows the addition of two sources of magnetization of the objects. The first source corresponds to the remanence magnetization resulting from the manufacturing or storage of the objects, and it can have any direction of magnetization. The second source of magnetization depends on the time the object stayed at the bottom of the water reservoir. This magnetization results from the fact that many munitions were sunk right after the second world war; therefore, they were magnetized by the Earth’s magnetic field. In total the following objects were modeled:

- UXO—Positive class:
  - cylinder with: diameter in range 75–200 mm, projectile length 350–650 mm
- non-UXO—negative class:
  - Too-small projectile to be dangerous: diameter in range 45–70 mm, projectile length 150–300 mm
  - Too-big projectile to be considered as UXO: diameter in range 250–400 mm, pipe length 750–1200 mm
  - a low height cylinder—(ex. simulating a manhole cover), diameter length 110–400 mm, thickness 20 mm
  - rectangular prism of length in range 400–1000 mm, width in range 50–200 mm and thickness in range 20–500 mm
  - rectangular cube of length in range 900–2000 mm, width 50–200 mm and thickness 50–200 mm

**Figure 14:**
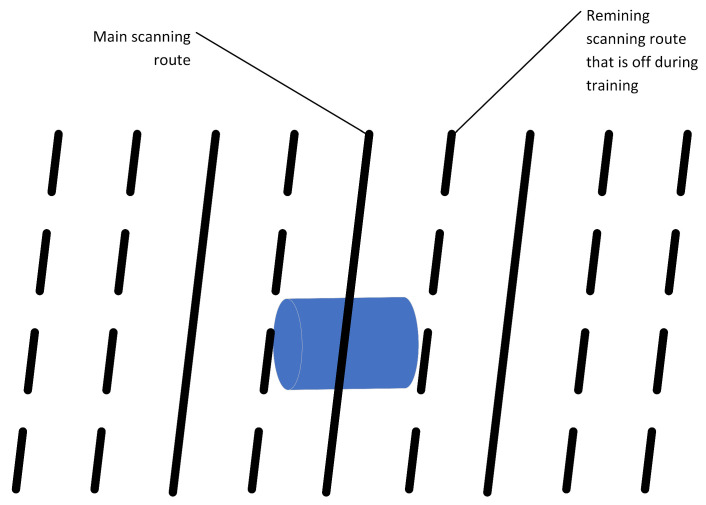
The parallel scanning routes. When building the model it can be assumed that only three routes were available with a different relation to the center of the object. Solid lines represent such a case, while a dashed line represents routes that were off during preprocessing of the final training set for the model.

In all cases, the magnetization was selected randomly in the range 0–0.3T, and it was assumed that the direction of magnetization is always consistent with the longest dimension of the object. This allowed us to run in total around 1600 simulations of these objects (digital twins). Then, for each digital twin, 30 random scanning routes were generated using the Matlab subroutine, where it was assumed that the height of the sensor’s path above the object is determined randomly within a range of 1 m to 3 m. The height of the probe is determined once for a single scan. Additionally, as indicated in the previous section, we added an element of randomness to the location of the sensor by adding a noise with a maximum amplitude of 10 cm. The direction of the scanning route was selected at random in the range 0–180 deg. This limitation results from the fact that, in the range 180–360 deg., the recorded signals are identical, and only flipped. Additionally, in the conducted simulations, it was assumed that a single scanning route consisted of a formation of nine virtual magnetometers, parallel to each other, as shown in Figure 14, where the fifth magnetometer always passes directly over the center of the object. The distance between the paths is 0.5 m. These nine parallel paths allow us to train and validate the prediction model in several scenarios. For example, by switching on and off particular routes, we can simulate scenarios when a particular magnetometer is scanning on the side of the UXO object instead of the center, or to identify the effect of the distance between virtual magnetometers on the prediction quality. In total, it resulted in around 48,000 training samples each consisting of nine parallel sensors recording the data. Therefore, the recorded data constitute a 4d tensor of 48,000 samples, nine probes, and 101 values of x, y, and z coordinates of the magnetic field.

The obtained dataset allows for the training of machine learning models in order to conduct UXO/non-UXO object classification. Although, the constructed dataset has several limitations. First, the dataset was obtained for particular geographical location determined by magnetic inclination and for magnetic field amplitude; in particular, these were the Earth’s magnetic induction $B_{e}=49.85\times 10^{-6}\;\left[T\right]$, and inclination 67${}^{\circ }$. These values correspond to the typical conditions in central Europe. For other areas, it is required to regenerate the data and retrain the machine learning model. Additionally, the model was tuned to conduct analysis, assuming the path of the probes is between 1 m and 3 m above the object, that is, the typical distance of the AUV from the bottom of the body of water. A limitation of this dataset is the set of non-UXO objects, but new ones can be easily added. When developing the digital twins of non-UXO objects, we tried to determine objects which surround possible shapes of the UXO object, so that the machine learning model could generalize acquired knowledge and reduce false-positive alarms. Additional limitations are the parameters that were used to generate UXO and non-UXO objects. For the identification of other objects, a new set of parameters is needed, and, again, the dataset needs to be regenerated and the model retrained.

## 7. Conclusions

In the manuscript, a process of creating a digital twin of the environment containing UXO and non-UXO objects was presented. The virtual environment was then tuned in terms of computational complexity and used to virtually scan the objects with virtual magnetometers. Such an environment has several advantages, it allows for easy collection of the training data for building machine learning models. It is safe, and does not require the supervision of the sapper, and allows for creating a massive training set in a range of several days. In our case, the calculations for creating the training set consisting of 46,000 samples took three days on a single two-processor machine (2xAMD EPIC processor and 512GB RAM), whereas, collecting the data for the physical experiments to collect eight scans took a single day.

In the manuscript, we have also shown how to simplify the model to speed up the calculations, without sacrificing the quality of the obtained results. The proposed solution significantly reduces the number of nodes of the mesh, which resulted in reducing the calculation time by 1/3. In general, we have shown that the approach of building virtual environments for generating data for training machine learning models is not only possible for vision problems, but can be also applied to other environments; however, careful tuning of the model is required to achieve results comparable to the one recorded with physical sensors. An example of such an environment is detecting the UXO objects in an underwater environment using magnetometer sensors. In the manuscript, we omit the problem of creating a machine learning model as it is the subject of another article and is out of the scope of this article.

## Figures and Tables

**Table 1:** Reference measures of mesh quality for the tested object.

SICN	Gamma	SIGE
0.6	0.5705	0.7469

**Table 2:** Real measures of mesh quality for the tested object.

Model	SICN	Gamma	SIGE
pipe	0.6	0.5705	0.7469
cylinder	0.5827	0.5543	0.7407

**Table 3:** Measures of mesh quality, number of nodes, and calculation time for the entire model.

Model	SICN	Gamma	SIGE	Nodes Number	Calculation Time
pipe	0.6692	0.6311	0.7543	173,863	00:40:36.4109984
cylinder	0.6946	0.6488	0.7517	78,488	00:14:37.7880462

**Table 4:** Measures of different mesh densities.

Parameter	Tube			Cylinder		
	LD	RD	HD	LD	RD	HD
Nodes tot.	98,234	173,863	696,208	74,288	78,488	125,804
Nodes in object	14,706	75,447	540,913	918	3700	19,227
Elements tot.	651,886	1,132,588	4,213,750	454,175	480,002	762,915
Elements in object	69,888	398,028	3,030,830	4128	18,576	103,800
Calculation time (hh:mm:ss)	00:17:59	00:40:36	02:12:42	00:12:36	00:14:37	00:26:38
SICN tot.	0.7119	0.6692	06429	0.6989	0.6946	0.7207
SICN for object	0.7025	0.6	0.6194	0.6299	0.5827	0.6771
Gamma tot.	0.668	0.6311	0.609	0.6521	0.6488	0.6727
Gamma for object	0.6663	0.5705	0.5883	0.5985	0.5543	0.6421
SIGE tot.	0.7615	0.7543	0.7559	0.7522	0.7517	0.7682
SIGE for object	0.7855	0.7469	0.7563	0.7543	0.7407	0.7769

**Table 5:** $R^{2}$ measures of different mesh densities.

Variant	Tube			Cylinder		
	LD	RD	HD	LD	RD	HD
N-S	0.946	0.960	1	0.961	0.981	1
E-W	0.989	0.996	1	0.993	0.998	1

**Table 6:** Results comparing the physical system with the digital twin.

UXO Orientation	Scanning Orientation	$R^{2}$	RMSE	MAE	MAX-MIN	ME	PSNR
N−S	N−S	0.984	$1.37\times 10^{-8}$	$1.04\times 10^{-8}$	$4.15\times 10^{-7}$	$1.25\times 10^{-8}$	29.62
E−W	N−S	0.950	$1.79\times 10^{-9}$	$1.49\times 10^{-9}$	$2.85\times 10^{-8}$	$1.60\times 10^{-8}$	26.06
NE−SW	N−S	0.985	$8.50\times 10^{-9}$	$7.14\times 10^{-9}$	$2.61\times 10^{-7}$	$8.04\times 10^{-9}$	29.74
Perpendicularly	N−S	0.984	$2.08\times 10^{-8}$	$1.64\times 10^{-8}$	$5.44\times 10^{-7}$	$1.94\times 10^{-8}$	28.35
E−W	E−W	0.968	$1.57\times 10^{-8}$	$1.46\times 10^{-8}$	$3.22\times 10^{-7}$	$1.55\times 10^{-8}$	26.23
N−S	E−W	0.872	$1.24\times 10^{-8}$	$1.15\times 10^{-8}$	$1.08\times 10^{-7}$	$4.85\times 10^{-9}$	18.79
NE−SW	E−W	0.968	$1.22\times 10^{-8}$	$1.09\times 10^{-8}$	$2.46\times 10^{-7}$	$1.18\times 10^{-8}$	26.11
Perpendicularly	E−W	0.993	$1.38\times 10^{-8}$	$8.90\times 10^{-9}$	$5.14\times 10^{-7}$	$1.17\times 10^{-8}$	31.43

**Table 7:** Comparison of error rates between the pipe and a cylinder substitute.

	UXO Orientation	Scanning Orientation	$R^{2}$	RMSE	MAE	MAX-MIN
Pipe	N-S	N-S	0.9849	$1.37\times 10^{-8}$	$1.04\times 10^{-8}$	$4.15\times 10^{-7}$
Cylinder	N-S	N-S	0.9843	$1.40\times 10^{-8}$	$1.07\times 10^{-8}$	$4.15\times 10^{-7}$

## Data Availability

The generated dataset is available at: http://bit.ly/uxodataset.
